# Editorial: Proton Therapy in Cancer Treatment: Clinical Evidence and Controversies

**DOI:** 10.3389/fonc.2021.791302

**Published:** 2022-01-28

**Authors:** Beate Timmermann

**Affiliations:** Department of Particle Therapy, University Hospital Essen, West German Proton Therapy Centre Essen (WPE), West German Cancer Center (WTZ), Germany, German Cancer Consortium (DKTK), Essen, Germany

**Keywords:** proton therapy, cancer, clinical evidence, treatment, clinical controversies

## Introduction

When speaking about proton therapy (PT), we typically walk between two lines. On the one hand we have a well-established radiotherapy modality representing a standard method for demanding entities like chordomas and chondrosarcomas of the base of skull and spine as well as uveal melanomas over the last 70 years already. More recently, also childhood cancer was understood to be an important target of PT. On the other hand, we have to acknowledge that there are still significant technical developments in progress to achieve the same standards in image guidance and robustness as are established in photon therapy for quite some time already. Therefore, the drive for further research but also controversies are typically arising either from the quest of getting PT into a broader clinical use for other (and more common) entities or from the ambition to successfully promote further technical progress. This present issue is truly reflecting the current status of proton therapy. It highlights the fact that the clinical role of PT in entities other than chordomas and eye tumours is definitely increasing. Still, this issue also clearly illustrates that physicists are still busy working on optimizing the technical aspects of proton beam therapy in areas like moving targets, robustness and range verification.

The Research Topic covers in total 11 articles on PT reflecting technical, biological and clinical questions. Any broader use of PT will be based on fundamental technical developments, helping to overcome current restrictions and addressing the challenges of a greater extent of uncertainties and the vulnerability of the static dose plan due to range uncertainties or to intrafractional interplay effects. If the superior physical characteristics of PT are supposed to lead to further clinical advantage, efficient control of uncertainties and better understanding of potential plan degradation have to be achieved. It is therefore conclusive that two studies within this Research Topic are focussing on lung tumours being the most challenging targets for proton beam therapy.

Wei et al. present an analysis of methods for mitigating interplay effects. Their retrospective study is focussing on the most modern proton modality, spot scanning proton beam therapy (i.e. pencil beam scanning [PBS]). Here it was used to deliver stereotactic body radiotherapy with repainting after robust 4-D optimization as a strategy to overcome uncertainties resulting from respiratory motion.

Another strategy to safely treat lung tumours by using particle radiation therapy (PRT) is presented by Emert et al., investigating motion-mitigation either by enhanced deep-inspiration breath hold (eDIBH) or high-frequency percussive ventilation (HFPV). Applicability, effectiveness, reproducibility, and subjects' acceptance were assessed and proven to be both effective and feasible.

Besides mitigating interplay effect, also improving lateral penumbra of pencil beam proton therapy is of major concern since one of the few disadvantages of active scanned versus passive scattered protons is the inferior lateral dose fall-off as the scanned pencil beams have typical widths larger than 1 cm. Therefore, investigations of adding apertures to PBS proton therapy is of major interest for the community. Bäumer et al. describe the clinical implementation of brass apertures in combination with active scanning proton delivery, proving that sharpening of the beam and improved sparing of organs at risk could be achieved.

The attractive dose distributions of proton fields can only be fully exploited in clinics if the uncertainties of the stopping-power estimation are well under control. Positron emission tomography (PET) range verification was understood as a method that can help to provide the confidence in PT for clinical applications. Zhang et al. implemented two verification methods of off-line PET verification and applied it to clinical breast cancer cases. It turned out, that both methods evaluated could quantify the accuracy of PT to the millimetre level.

Very much alike a summary of the most relevant current issues, Mazal et al. list various limitations of protons in clinical practice, like uncertainties in range, lateral penumbra, deposition of higher LET outside the target, organ movements and eventually cost. In their review, interesting and innovative methods to mitigate those pitfalls are proposed to be further studied, such as “FLASH” irradiation, mini-beams, rotational techniques and, gantry-less treatment approaches.

He also highlights the importance of improving the biological understanding of particle therapy. Therefore, studies like presented by Suckert et al. seem very much appropriate. The authors describe a preclinical model to reveal biological mechanisms caused by precise high-dose proton irradiation of a brain sub-volume and try to derive a dose–response model in order to optimise future experiments and to potentially support evaluation of brain toxicity after proton therapy.

In general, in the field of medicine, but particularly in the field of PT, focussing research on clinical needs is of crucial importance, thus delivering positive impact on improved individual patient treatment. Even with fantastic instruments being accessible, we may not always use them in the best way. The study from Sha et al. reflect how new treatment techniques could be realised, by adopting long-used historical strategies. Here, the authors have managed to improve cardiac sparing by separating the PTV of the right and left lung rather than using one PTV on both lungs.

In proton beam therapy, the body of clinical evidence is rapidly growing, also caused by the increasing number of facilities and therefore greater capacity to perform clinical studies. Hence, we could include a number of clinical papers in this Research Topic, both reviews and original studies.

Jazmati and colleagues report on their findings on neuroblastoma from a large prospective paediatric proton registry. In their study, PT was well tolerated and effective in young children with neuroblastoma. The authors still remind us, that any RT has to be regarded in the view of the multidisciplinary treatment regimen and that particularly in very young children open questions remain to be studied in order to balance risks for adverse events against gain in tumour control.

Doyen et al. are dealing with a very different cohort. They reviewed 127 patients with both benign and malignant tumours retrospectively, having received high dose rate pulsed PT with 10 Gy per second. The authors could not reveal a major decrease in acute and subacute toxicity. However, they discuss the need for further investigation and the chances arising from “FLASH” radiotherapy for patients as it was suggested by preclinical studies.

Our Research Topic also includes one clinical review. Weber et al. summarise findings in PT for recurrent and primary meningioma. They conclude that PT is routinely used for treatment of meningiomas and that it may not be limited to volumetrically challenging tumours, non-benign histology or for the re-irradiation of recurrent and progressive tumours. They still state, that some scenarios may need to be discussed on a case by case basis also taking into account age and tumour grading.

Interestingly, the question of patient selection on a case by case basis is also addressed in this Research Topic from a very different, computational perspective. As the area of artificial intelligence and big data has further evolved over time, machine learning may help clinicians in making qualified decisions. Qiu et al. present an original study on predicting outcome for high grade glioma (HGG) patients, by using machine learning to provide a tool of reference for counselling PBS as a treatment option for HGG.

## Concluding Remarks

We are convinced, that the ambition and eagerness to answer the open questions in proton beam therapy - reflected by this Research Topic – will finally lead to a wider PT offered to a higher number of cancer patients in critical scenarios. Besides improving survival rates, also lowering the price of survival is of particular importance for cancer patients. Both are understood as being the fundamental aims when incorporating PT into a wider range of oncological strategies. Still, as the financial effort to offer PT is significantly higher when compared to photon radiotherapy, it will be crucial to gain further clinical evidence in order to convince policy makers.

We are eager to follow the future evolution of PT in science and clinics.

## Author Contributions

The author confirms being the sole contributor of this work and has approved it for publication.

## Conflict of Interest

The author declares that the research was conducted in the absence of any commercial or financial interest.

## Publisher’s Note

All claims expressed in this article are solely those of the authors and do not necessarily represent those of their affiliated organizations, or those of the publisher, the editors and the reviewers. Any product that may be evaluated in this article, or claim that may be made by its manufacturer, is not guaranteed or endorsed by the publisher.

